# Supplementary material for: Transcriptome-Based WGCNA Analysis Reveals the Mechanism of Drought Resistance Differences in Sweetpotato (Ipomoea batatas (L.) Lam.)
Source: Int J Mol Sci. 2023 Sep 21;24(18):14398. doi: 10.3390/ijms241814398 (PMC10531967; doi:10.3390/ijms241814398)
Supplement: Supplementary file 1 [file ijms-24-14398-s001.zip › Figures S1-S6.pdf]

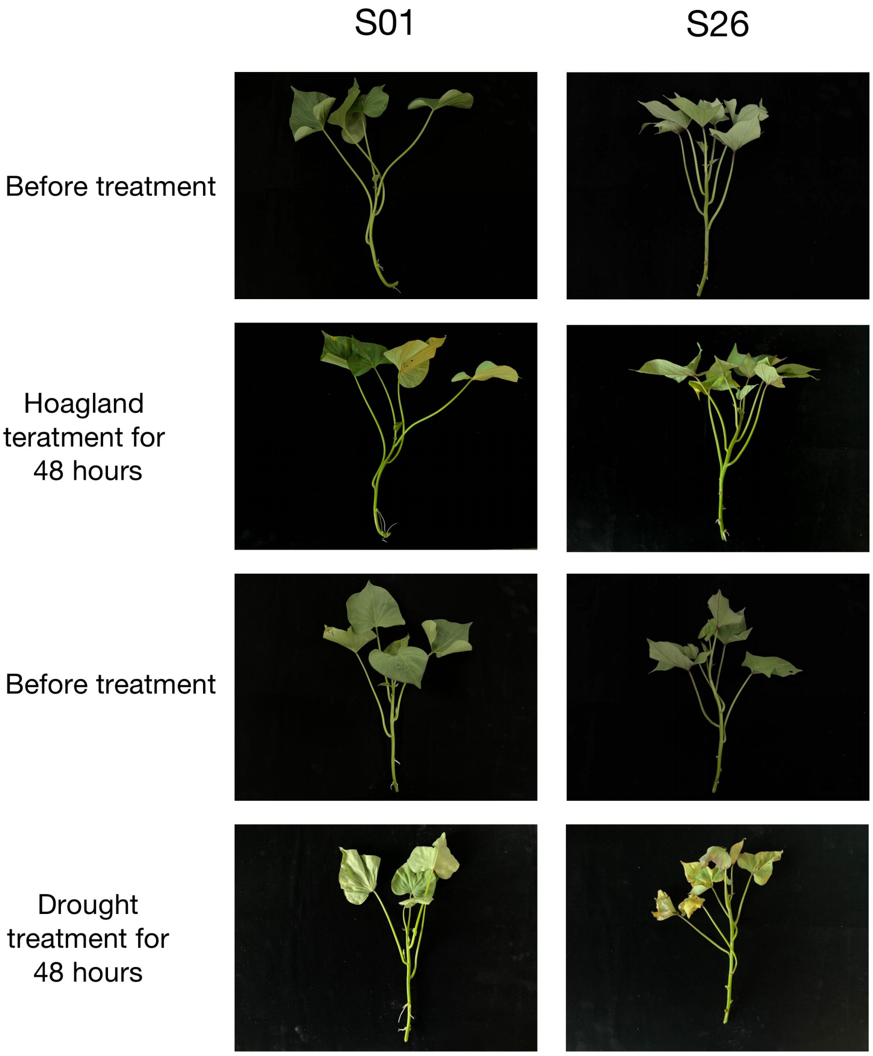

Figure S1. Phenotype changes of S01 and S26 drought treatment groups and control groups

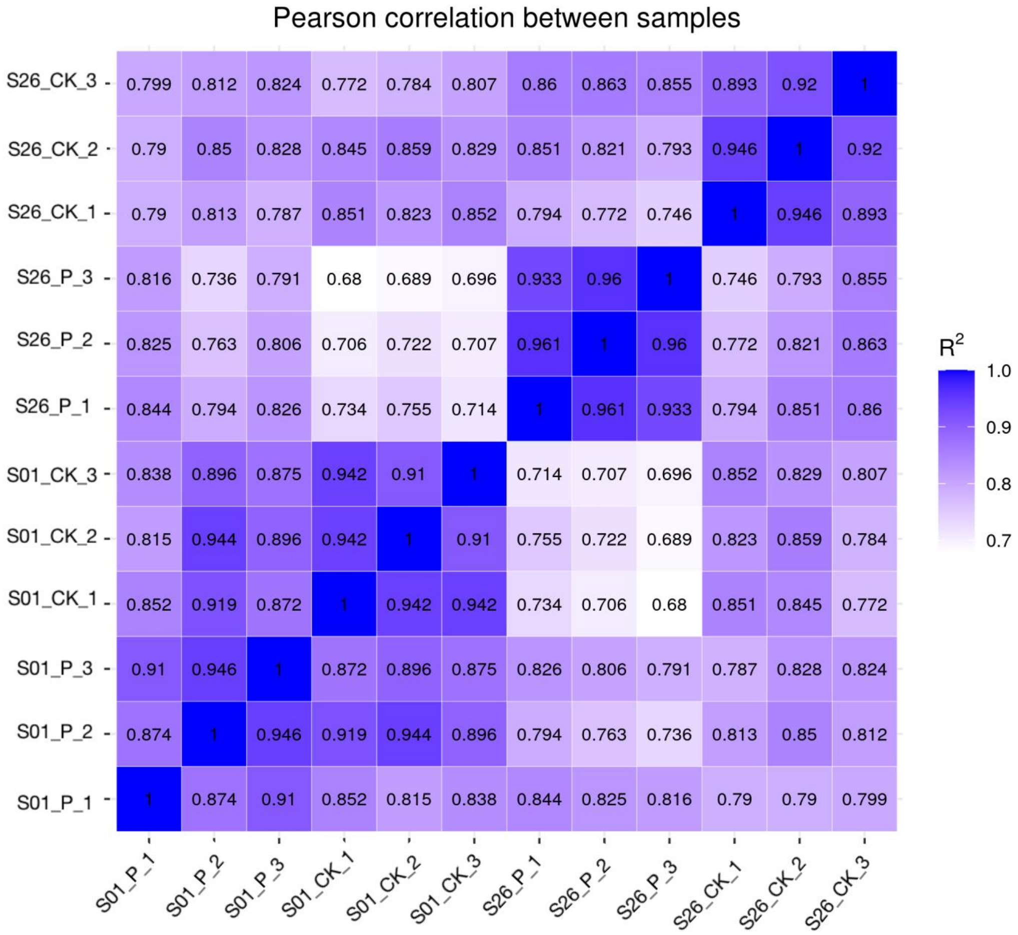

Figure S2. Correlation analysis of sample gene expression levels

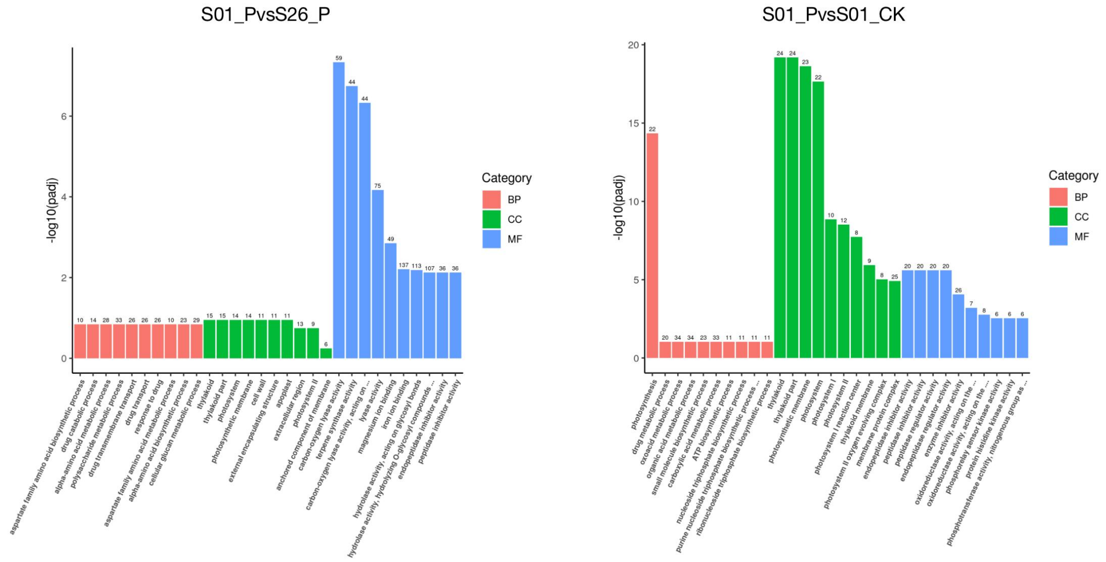

Figure S3. Differential gene GO enrichment analysis results. BP=biological process,CC=cellular component;MF=molecular function

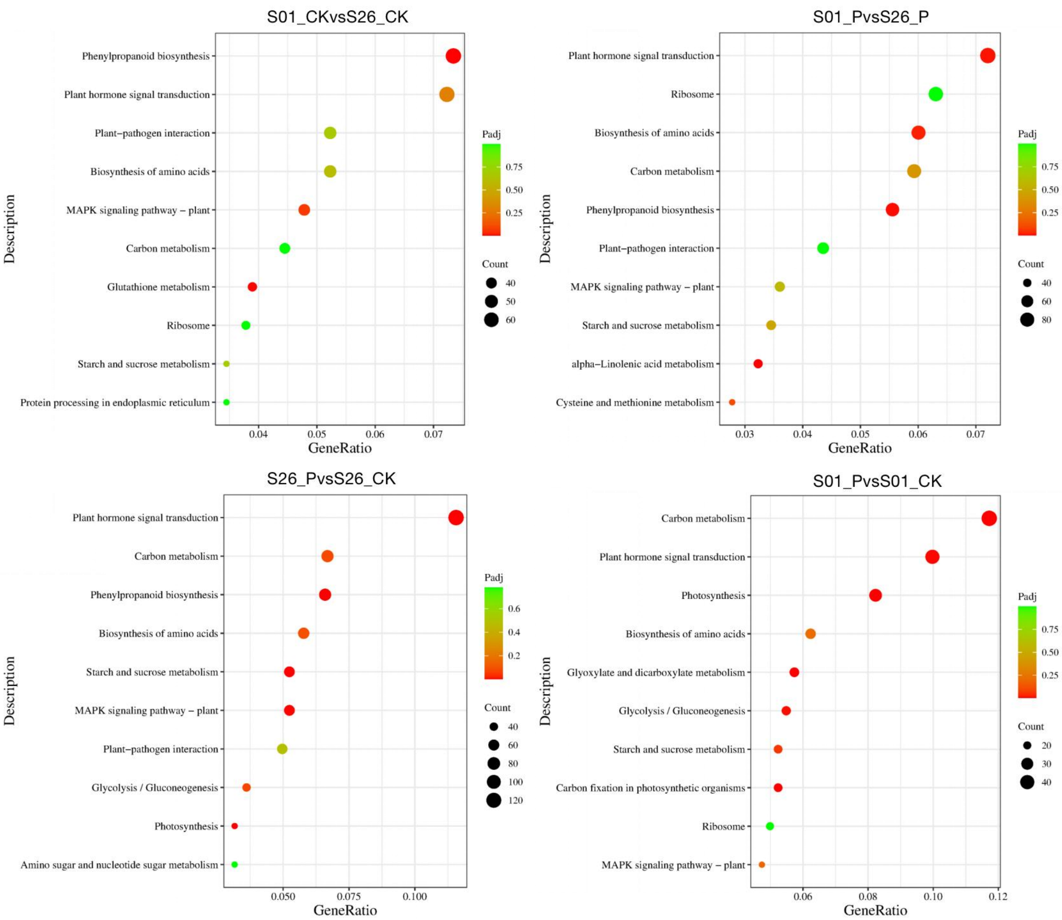

Figure S4. KEGG metabolic pathway analysis of DEGs. The size of the circles represents the number of genes annotated to this pathway

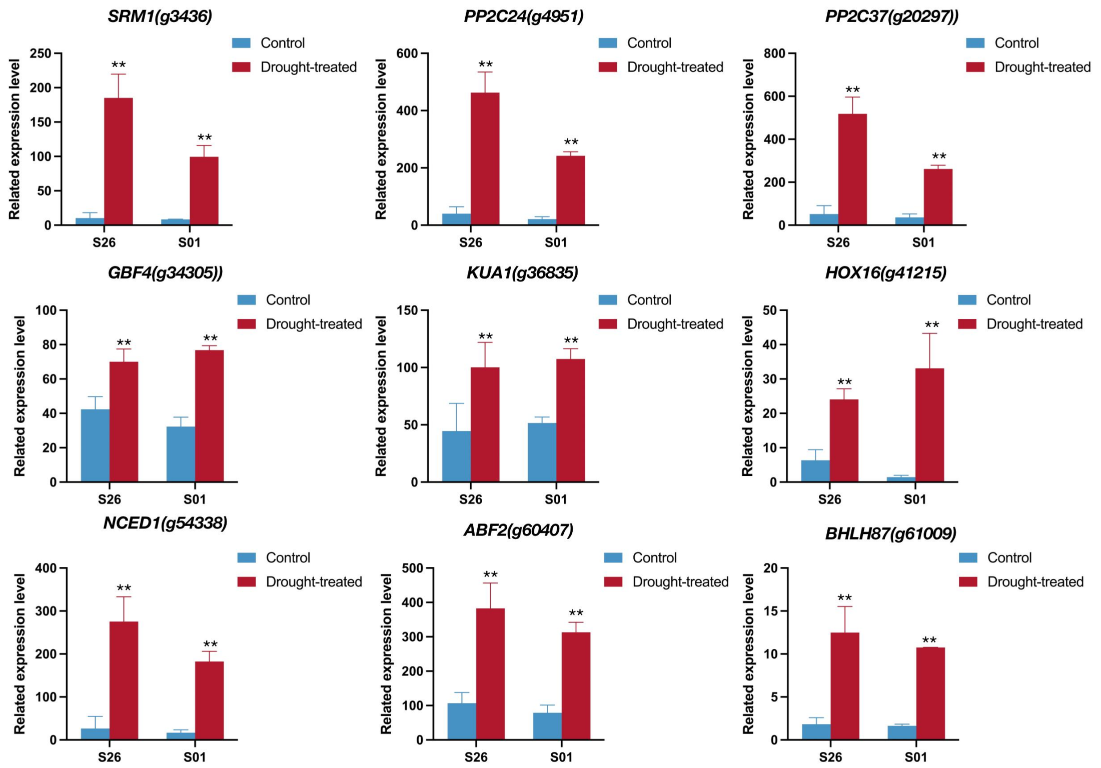

Figure S5. RNA seq data gene expression levels. ** indicates that the different physiological index between control group and experimental group was significant at *P* value < 0.01.

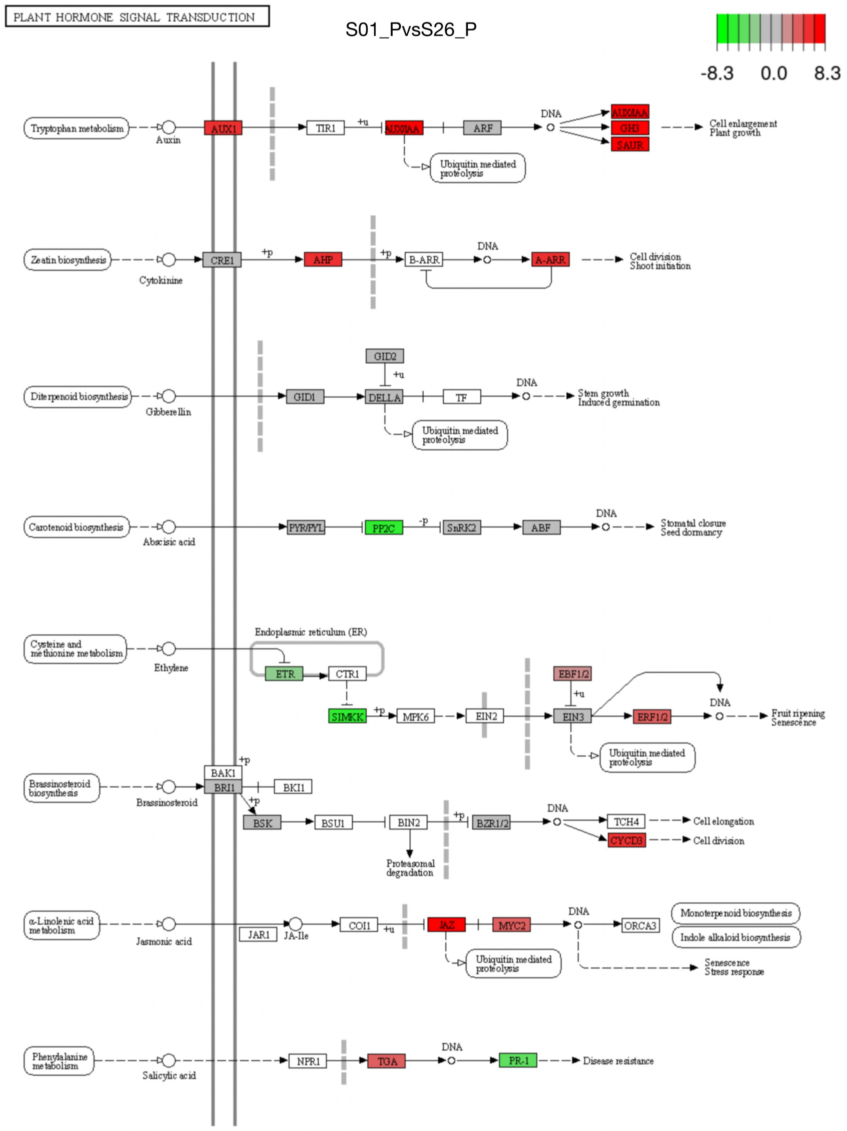

Figure S6. Plant hormone conduction pathway diagram
